# Severe Liver Damage in an Obese Patient: Onset of Celiac Disease or Overlap Syndrome?

**DOI:** 10.3390/diagnostics14161832

**Published:** 2024-08-22

**Authors:** Gabriela Ghiga, Laura Otilia Boca, Elena Cojocaru, Iuliana Magdalena Stârcea, Elena Țarcă, Ana Maria Scurtu, Maria Adriana Mocanu, Ileana Ioniuc, Mihaela Camelia Tîrnovanu, Laura Mihaela Trandafir

**Affiliations:** 1 Department of Mother and Child, Faculty of Medicine, University of Medicine and Pharmacy “Grigore T Popa”, 700115 Iasi, Romania; gabriela.ghiga@umfiasi.ro (G.G.); magdalenastarcea@gmail.com (I.M.S.); adriana_baltag@yahoo.com (M.A.M.); ileana.ioniuc@umfiasi.ro (I.I.); mihaela.tirnovanu@umfiasi.ro (M.C.T.); laura.trandafir@umfiasi.ro (L.M.T.); 2 “Saint Mary” Emergency Hospital for Children, 700309 Iasi, Romania; elena2.cojocaru@umfiasi.ro (E.C.); tarca.elena@umfiasi.ro (E.Ț.); a_m_scurtu@yahoo.com (A.M.S.); 3 Department of Morphofunctional Sciences, Faculty of Medicine, University of Medicine and Pharmacy “Grigore T Popa”, 700115 Iasi, Romania; 4 Department of Pediatric Surgery, Faculty of Medicine, University of Medicine and Pharmacy “Grigore T Popa”, 700115 Iasi, Romania; 5 “Cuza Voda” Obstetrics-Gynecology Clinic Hospital, 700038 Iasi, Romania

**Keywords:** celiac disease, liver disease, hepatitis, autoimmune hepatitis, gluten-free diet

## Abstract

Celiac disease (CeD) is an enteropathy caused by the complex interaction between genetic, environmental, and individual immunological factors. Besides the hallmark of intestinal mucosal damage, CeD is a systemic disorder extending beyond the gastrointestinal tract and impacting various other organs, causing extraintestinal and atypical symptoms. The association between CeD and liver damage has been classified into three main categories: mild and asymptomatic liver injury, autoimmune liver injury, and liver failure. We present a case of severe liver damage with cirrhotic evolution in an obese 12-year-old boy who had been admitted due to generalized jaundice and localized abdominal pain in the right hypochondrium. In the course of investigating the etiology of severe liver disease, toxic, infectious, metabolic, obstructive, and genetic causes were excluded. Despite the patient’s obesity, a diagnosis of CeD was established, and in accordance with autoimmune hepatitis (AIH) criteria, the patient was diagnosed with autoantibody-negative AIH associated to CeD.

## 1. Introduction

CeD is a complex enteropathy caused by the interaction between genetic, environmental, and individual immunological factors. CeD is induced by the ingestion of gluten in genetically predisposed individuals, representing a permanent intolerance to gluten. It leads to villous atrophy in the small intestine and typically resolves after the strict elimination of gluten from the diet [1,2]. Due to widely available diagnostic tools, clear diagnostic algorithms, and increased awareness of the disease, its diagnosis rate is continuously increasing, affecting approximately 0.7–1% of the general population [3,4].

The phenotypic expression of CeD includes a wide variety of manifestations, ranging from typical gastrointestinal forms to atypical or asymptomatic forms [5,6]. The primary manifestation of CeD is represented by small intestine injury, causing malabsorption syndrome with, consequently, malnutrition and gastrointestinal symptoms, such as diarrhea, vomiting, constipation, diffuse abdominal pain, and abdominal meteorism [7]. Besides the hallmark of intestinal mucosal damage, CeD is a systemic disorder extending beyond the gastrointestinal tract and impacting various other organs, causing extraintestinal and atypical symptoms [1].

Several liver abnormalities associated with CeD have been reported [8]. It has been hypothesized that CeD has the potential to cause hepatic injury itself, but it can also influence the clinical dynamic of concurrent chronic liver diseases [2]. Liver involvement in CeD typically presents as mild and reversible increase in transaminases, but it can also present as AIH, metabolic dysfunction-associated steatotic liver disease, cholestatic liver disease, primary sclerosing cholangitis, and primary biliary cirrhosis, and in rare instances, it can progress to liver failure [7,9].

The most common hepatic manifestation of CeD is represented by elevated aminotransferase levels, occurring in 9–14% of cases [7]. Many studies have shown that the elevation in aminotransferase levels correlates directly with duodenal mucosal damage, malabsorption, and serum tissue transglutaminase levels [10]. Following the adoption of a gluten-free diet (GFD), aminotransferase levels usually normalize within approximately six to twelve months [11]. Strong evidence indicates that primary biliary cirrhosis and AIH are linked to CeD at a rate exceeding random occurrence. This association might be due to the shared genetic combinations that encode HLA class II molecules on chromosome 6, which are common to both CeD and AIH [2,12].

In the following paragraphs, we present a case of severe liver damage with cirrhotic evolution in a 12-year-old boy who had been admitted due to generalized jaundice and localized abdominal pain in the right hypochondrium.

## 2. Case Report

A 12-year-old male patient was urgently admitted to our clinic due to generalized jaundice and localized abdominal pain in the right hypochondrium. There was no reported family history of liver, gastrointestinal, or autoimmune disorders.

Upon admission, the patient presented with moderately affected general condition, reduced appetite, absence of fever, obesity (height = 182 cm and Z index = +3.71, weight = 92.5 kg and Z index = +2.94, BMI= 27.9 kg/m^2^, situated on the 98th percentile, Z index = +2.66), jaundiced skin and sclerae, a painful abdomen in the right hypochondrium, hyperchromic urination, and the lower edge of the liver palpable 3 cm below the costal margin.

An initial biological assessment revealed mild neutropenia, significant eosinophilia, and mild normochromic normocytic anemia, in the presence of inflammatory syndrome. The peripheral blood smear did not reveal any pathological abnormalities. Hepatic function tests highlighted a significant increase in AST (12.8 times the normal value), ALT (14.8 times the normal value), direct bilirubin (20.8 times the normal value), and gamma-glutamyl transferase (GGT) (7.2 times the normal value) and a slightly elevated INR, while cholinesterase, fibrinogen, aPTT, and prothrombin time were within normal limits. Additionally, total proteins were within normal limits, while protein electrophoresis revealed only a slight increase in gamma globulins. Serum IgE levels were notably elevated (4.48 times the normal value).

Toxic causes were excluded through repeated discussions with the parents and the patient. Additionally, infectious causes were ruled out through testing for anti-HCV antibodies, HbS antigen, syphilis TP, PRP, Quantiferon, Epstein–Barr virus antibodies, cytomegalovirus antibodies, anti-toxoplasma antibodies, anti-SARS-CoV-2 antibodies, and anti-toxocara canis antibodies, all of which were within normal limits. 

Furthermore, some metabolic pathologies that could explain the symptoms and biological abnormalities were ruled out: serum ceruloplasmin, copper levels, urinary copper excretion, serum iron and ferritin, alpha-1 antitrypsin—all within normal limits—excluding hemochromatosis, alpha 1 antitrypsin deficiency, and Wilson’s disease. Additionally, thyroid function was found to be within normal limits.

Anti-LKM 1, anti-MPO, and anti-ASMA antibodies were measured in order to exclude AIH, all of which were within normal values. In order to rule out other autoimmune conditions, a comprehensive ANA panel was conducted (including Hep2, dsDNA, ssDNA, Sm, Rnp/Sm, ssa-Ro60m, SSB-La, and Scl-7), with all results within normal limits.

Following genetic consultation, genetic testing was advised and carried out in order to rule out storage disorders (Pompe, Niemann–Pick, Gaucher, and mucopolysaccharidoses) and cystic fibrosis, all with normal results. A bone marrow examination was performed, which did not reveal any pathological features.

Abdominal ultrasound revealed a hyperechogenic liver and an enlarged spleen. Under treatment with arginine, ursodeoxycholic acid, and acetylcysteine, the biological evolution was slowly favorable, prompting the continuation of further investigations.

An abdominopelvic computed tomography (CT) scan was performed, and it revealed significant hepatosplenomegaly (right hepatic lobe diameter—8.7 cm, left hepatic lobe diameter—8.2 cm, craniocaudal diameter of the spleen—18.5 cm) and adenopathy in the hepatic, celiac, and portocaval regions. A moderately hypodense band with densities ranging between 30 and 50 Hounsfield units (HU) was observed, lacking significant contrast enhancement along the border of the portal vein at the hilum and its branches. This raised suspicion of periportal fibrosis. A magnetic resonance cholangiopancreatography (MRCP) was performed, which confirmed the global hepatomegaly and grade II/III splenomegaly, but it did not reveal any evidence of obstructive changes.

**Figure 1:**
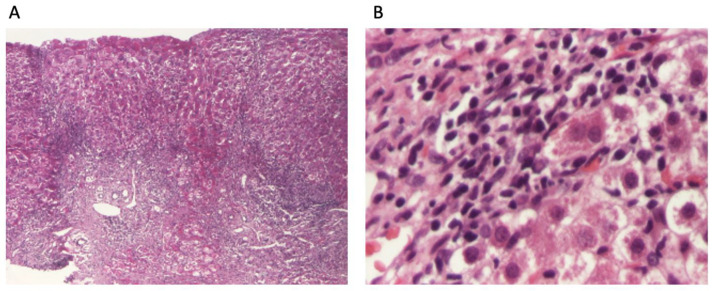
Liver biopsy: (**A**). overview of liver biopsy with portal and periportal necro-inflammatory activity (HE stain ×40); (**B**). abundant inflammatory infiltrate with mononuclear cells and relatively numerous granulocytes, with interface hepatitis aspects and hepatocytes with granulo-vacuolar degeneration (HE stain ×400).

**Figure 2:**
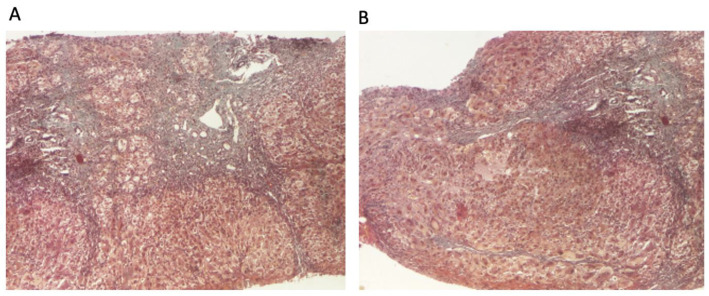
Liver biopsy: (**A**) hepatic architecture distorted by zones of variable portal and periportal fibrosis with bile duct proliferation (Szekely trichrome stain ×40); (**B**) signs of ongoing hepatitis and cirrhotic evolution (portal and periportal mononuclear inflammation, interface hepatitis, and fibrosis) (Szekely trichrome stain ×40).

The patient continued to receive the aforementioned treatment without improvement in liver cytolysis and cholestatic syndrome. Due to persistent liver cytolysis syndrome, a liver biopsy was also performed, revealing in Hematoxylin–Eosin stain (HE) abundant inflammatory infiltrate with mononuclear cells and frequent granulocytes, interface hepatitis (piecemeal necrosis lesions), and occasional neo-biliary canaliculi within the inflammatory portoportal septa (Figure 1A,B). Szekely trichrome staining highlighted evident fibrosis in the porto-biliary spaces with a tendency to progress into septa, resulting in advanced massive chronic hepatitis with cirrhotic evolution (Figure 2A,B).

Given the diagnosis of chronic hepatitis with cirrhotic evolution, further investigations were conducted to determine the etiology and specific treatment. Although obesity in this patient made CeD an unlikely initial diagnosis, the unfavorable clinical evolution without a clear etiology required the measurement of anti-tissue transglutaminase IgA antibodies, which were elevated (7.2 times the normal value), along with positive anti-endomysium antibodies.

**Figure 3:**
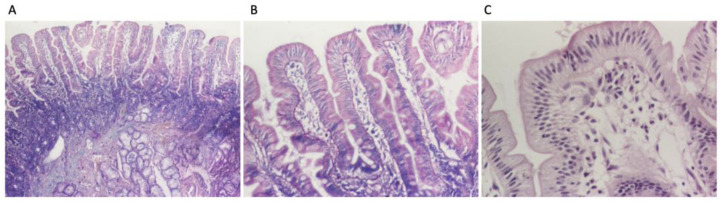
Duodenal biopsies: (**A**) intestinal villi with normal architecture and moderate inflammatory infiltrate in lamina propria (HE stain ×40), (**B**) less than 30 intraepithelial lymphocytes per 100 enterocytes (HE stain ×100); (**C**) intestinal villi with less than 30 intraepithelial lymphocytes per 100 enterocytes (HE stain ×200).

Following the diagnostic algorithm for celiac disease proposed by ESPGHAN in 2020, upper gastrointestinal endoscopy with intestinal biopsies from the distal duodenum and from the duodenal bulb was deemed necessary in this case. The investigation revealed a congestive purpuric and macro-nodular appearance in the antral region, leading to the diagnosis of congestive purpuric gastritis. A histopathological examination indicated moderately chronic active duodenitis with below 30 intraepithelial lymphocytes per 100 enterocytes, corresponding to Marsh 0, and mild chronic gastritis which was Helicobacter pylori negative (Figure 3A–C). Anti-tissue transglutaminase antibody testing from duodenal mucosa could not be performed due to technical reasons. Due to the high suspicion for CeD, a genetic test was performed, which was positive (HLA DQ 2.5 cis positive). Considering all elements, the diagnosis of CeD was confirmed.

At this stage, the differential diagnoses considered were hepatitis as a form of onset of CeD, seronegative autoimmune hepatitis associated with CeD as second autoimmunity, or just metabolic dysfunction-associated steatohepatitis (MASH) as a completely separated disease associated to CeD. According to the complete diagnostic criteria for AIH, the patient had a score of 16 before treatment and a score of 18 after treatment initiation, a score which is highly suggestive of the diagnosis of AIH. According to the simplified criteria for AIH, the patient has a score of six which probably corresponds to AIH. The patient’s score was based on the increased gamma globulin levels, absence of viral markers, lack of a positive history for hepatotoxic drugs and alcohol, presence of celiac disease as a second autoimmune pathology, and the presence of specific anatomical–pathological changes in AIH, particularly piecemeal necrosis (interface hepatitis).

**Figure 4:**
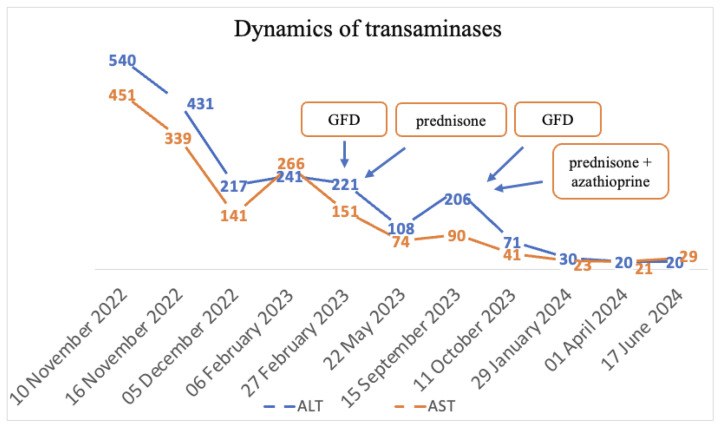
Dynamics of transaminases.

**Figure 5:**
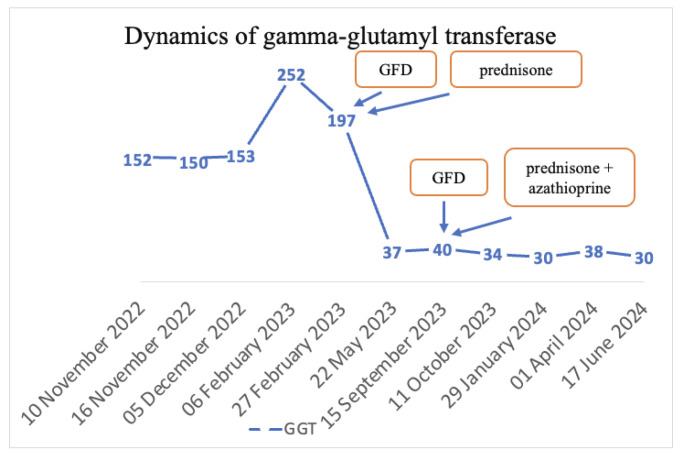
Dynamics of GGT.

The final diagnoses were seronegative chronic autoimmune hepatitis with cirrhotic evolution, CeD, and obesity. A GFD was initiated, and prednisone was introduced as treatment, but the patient was non-compliant with the diet, treatment, and periodic check-ups. Non-compliance impacted the biological evolution, and until September 2023, the transaminases values remained elevated. Since September 2023, discussions about the importance of diet and treatment have resumed. Consequently, a lifelong GFD and prednisone for 3 months with gradual dose reduction in association with azathioprine were introduced. Subsequently, with the introduction of prednisone and azathioprine therapy and strict adherence to the GFD, transaminase levels gradually decreased until normalization and both anti-tissue transglutaminase and anti-endomysium antibodies normalized (Figure 4 and Figure 5). Regarding the ultrasound findings, the liver appears within normal parameters, but there is a mild splenomegaly.

The patient is currently in remission, as indicated by normal levels of transaminases, gamma globulins, and specific antibodies for AIH (which have consistently been negative). According to European guidelines, azathioprine treatment should be continued for 3 years [13]. Consequently, the patient remains on a GFD and continues to receive azathioprine. If the clinical, biological, imaging, and histological evolution remains favorable and if the patient maintains remission after 3 years of azathioprine therapy, this medication will be discontinued, while the GFD will be maintained for life. An important aspect, considering that the patient is obese, consists of the implementation of nutritional counseling aimed at achieving weight loss and maintaining a healthy weight thereafter. This involves creating a personalized diet plan, monitoring the progress regularly, and providing ongoing support to ensure adherence to dietary recommendations. Effective management of weight is crucial for overall health and optimizing the patient’s response to ongoing treatments.

## 3. Discussion

**Figure 6:**
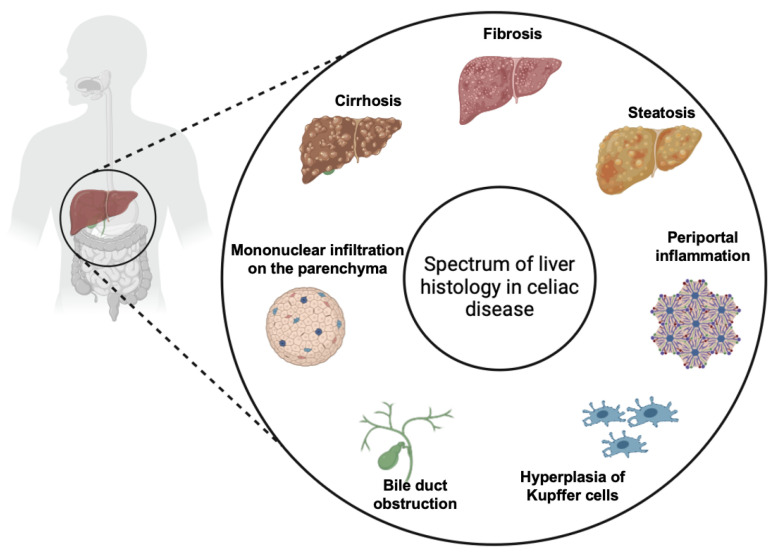
Spectrum of liver histology in CeD [2,15]. Created with Biorender.com.

While CeD primarily affects the small intestine, it is increasingly recognized for its wide array of extraintestinal manifestations, which can complicate diagnosis and management. Extraintestinal symptoms are common in patients with CeD, occurring in 20–30% of cases, and liver involvement is well documented in the medical literature [14]. The association between CeD and liver injuries, first described in 1977, has been classified in three main categories: mild and asymptomatic liver injury characterized by non-specific histological alterations which are usually reversible on GFD (Figure 6), autoimmune liver injury including AIH, autoimmune overlap syndrome, primary sclerosing cholangitis and primary biliary cirrhosis which usually require immunosuppressive therapy associated to GFD, and liver failure which usually is reversible on GFD [9]. However, two principal forms of liver injury in CeD are recognized: the non-specific celiac hepatitis and autoimmune forms. It is not yet very well clarified if the two forms are completely different diseases or if they are only two different stages of the same disease (“celiac liver disease”) [14]. Therefore, the clinical presentation of liver disease in CeD varies from an asymptomatic form, with mild hypertransaminasemia, to more severe forms associated with liver fibrosis, cirrhosis, and liver failure [2]. Elevated transaminases are a common feature in CeD, with hypertransaminasemia found at the moment of diagnosis in 15–61% of patients [14,15]. Furthermore, celiac patients have a higher risk of developing liver disease and cirrhosis compared to the general population [15,16]. On the other hand, CeD is found in 9% of patients who were diagnosed with chronic hypertransaminasemia [16].

**Figure 7:**
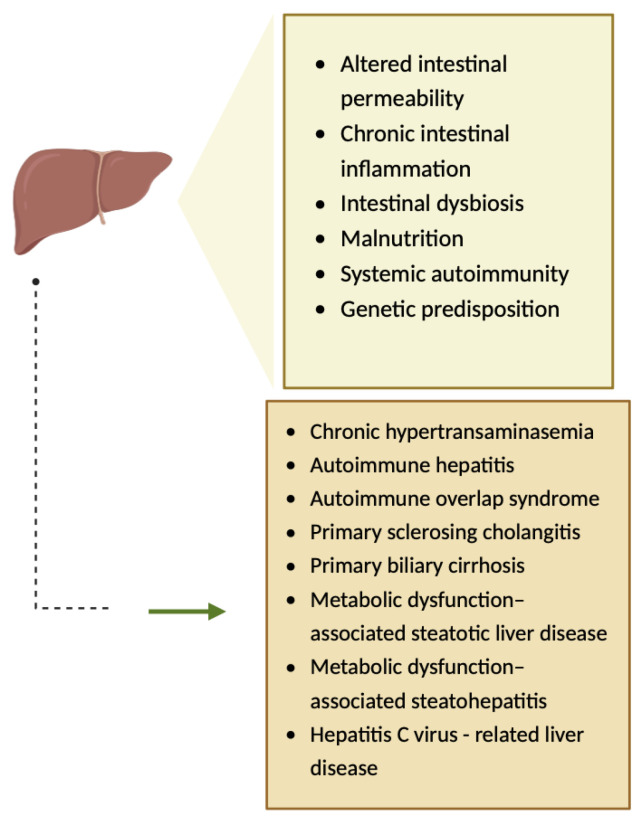
The pathogenesis of hepatic injuries associated with CeD [10]. Created with Biorender.com.

The pathogenesis of hepatic injuries associated with CeD is complex, involving several mechanisms such as altered intestinal permeability (Figure 7) [10,17,18]. Gliadin is responsible for the increased gut permeability, as it binds to CXCR3 (chemokine receptor), and consequently, MyD88-dependent zonulin is released, a process that allows toxins to reach the liver via portal circulation, leading to hepatic inflammation [19,20]. Furthermore, chronic intestinal inflammation, intestinal dysbiosis, malnutrition, systemic autoimmunity, and genetic predisposition have been hypothesized to be involved in the pathogenesis of hepatic injuries associated with CeD (Figure 7) [17,18,20].

The main diagnostic considerations in our case were autoantibody-negative autoimmune hepatitis associated to CeD, celiac hepatitis as a form of onset of CeD, and MASH associated to CeD.

Metabolic dysfunction-associated steatotic liver disease (MASLD), including MASH, is the most common cause of chronic liver injury, making it the primary diagnosis considered for an obese patient with a hyperechogenic liver via abdominal ultrasound. However, the acute onset characterized by abdominal pain and generalized jaundice, persistent cholestasis and hepatocellular injury, and elevated gamma globulins raised the suspicion of another condition, requiring the exclusion of other etiologies. Furthermore, the histological findings from the liver biopsy excluded MASLD as the sole cause of liver damage in this patient.

Celiac hepatitis is characterized by liver injury, biologically expressed as abnormal liver tests which can be associated with non-specific histological changes (non-specific reactive hepatitis) observed in liver biopsy in patients diagnosed with CeD. The hepatic involvement typically resolves after 6–12 months of following the gluten-free diet, which is the cornerstone of celiac disease therapy [2,21]. However, there are rare cases in which celiac hepatitis evolutes into severe liver disease with cirrhosis, fibrosis, or even liver failure [22,23].

AIH is a severe autoimmune liver disease characterized by chronic and progressive inflammation mediated by the immune system, leading to cirrhosis and liver failure. The incidence of AIH ranges from 0.85 to 1.68 per 100,000 person-years, with a notable predilection for women. Its pathogenesis is very complex, involving genetic predisposition, dysregulation of immune responses, and environmental factors. AIH is associated with various extrahepatic autoimmune conditions (autoimmune thyroiditis, diabetes, inflammatory bowel disease, and CeD), with a higher prevalence compared to the general population [24].

Inflammation is a defining feature of both AIH and CeD, manifesting in various forms and degrees of severity. The underlying inflammatory mechanisms in these conditions involve genetic predispositions, T-cell-mediated immune responses, and environmental triggers, establishing inflammation as a common characteristic in these autoimmune conditions [25,26]. In both diseases, inflammation is caused by T-cell-mediated immune responses, characterized by an imbalance between pro-inflammatory and regulatory T cells. In AIH, CD4+ T cells target hepatocytes, leading to chronic inflammation and fibrosis and, in CeD, T cells target the intestinal cells [8,26,27].

Increased intestinal permeability in celiac patients leads to increased circulation of anti-tissue transglutaminase antibodies, which may contribute to liver inflammation, as these antibodies interact with diverse antigens in liver and other tissues, forming new antigenic targets that can exacerbate liver damage [25]. Pro-inflammatory cytokines, particularly those associated with Th1 and Th17 cells, play a pivotal role in the pathogenesis of both AIH and CeD, contributing to tissue damage and also to the chronicity of the diseases [25]. Both diseases share common genetic predispositions, particularly involving HLA class II molecules such as DQ2, DR3, and DQ4, which may explain the frequent co-occurrence of these diseases [25,28].

In their study, Pezzato et al. included 166 individuals diagnosed with AIH who were followed for a median of 63 months. Their conclusions showed that CeD is associated with AIH in 5.4% of cases. Furthermore, patients with both CeD and AIH experienced less severe disease progression and a higher rate of successful withdrawal from immunosuppressive therapy compared to individuals with only AIH [29]. Similar observations were reported by Nastasio et al. in 2013, who proved that patients diagnosed with AIH and CeD who adhere to a GFD appear to have a reduced likelihood of relapse after discontinuing immunosuppressant therapy, in contrast to AIH patients without CeD [30].

Over recent decades, there has been a notable shift in the clinical presentation and epidemiology of CeD, reflecting changes in diagnostic practices, awareness, and possibly environmental factors. There has been observed a shift from classical to non-classical and subclinical presentations, as the proportion of patients with non-classical or subclinical CeD has increased from 14.8% before 1985 to 51.6% after 2010 [31]. Furthermore, in a study conducted in Italy involving 770 celiac patients, the authors found that the onset of CeD was symptomatic in 610 patients (representing 79% of patients), with 210 exhibiting a classical phenotype and 400 a non-classical phenotype. Meanwhile, 160 patients (21%) experienced a subclinical phenotype. Between 1998 and 2007, the classical, non-classical, and subclinical phenotypes were identified in 47.2%, 43.1%, and 9.7% of celiac patients, respectively. In contrast, from 2008 to 2012, the most common clinical phenotype was non-classical (58.2%), followed by subclinical (28.5%) and classical (13.3%). The most common extraintestinal manifestations comprised osteopenia and osteoporosis in 52% of cases, anemia in 34% of cases, cryptogenic hypertransaminasemia in 29% of cases, and recurrent miscarriages in 12% of cases [32].

Garapazzi et al. found out similar results, with a decrease in the prevalence of typical forms of CeD, an increase in the incidence of silent forms of CeD, and a decrease in subtotal villous atrophy with an increase in partial villous atrophy [33].

Overweight and obesity seem to be more prevalent at the time of diagnosis in patients with CeD than previously reported, with the prevalence varying from 8.8% to 20.8% [34]. The relationship between HLA DQ2 and obesity has been a subject of investigation, particularly in the context of children at genetic risk for type 1 diabetes. The study conducted by Yang et al. concluded that the HLA-DQ 2.2 genotype may predispose children aged 2-4 years to higher obesity risk, with a significant association observed at age 4 [35]. These findings suggest a complex relationship between HLA-DQ genotypes and obesity, but, as far as we know, there is no research specifically on the association between HLA DQ2.5 and obesity, warranting further investigation.

Regarding the CeD diagnosis in this obese patient, the specific antibodies were positive (less than 10 times the normal values), and an upper digestive endoscopy with duodenal biopsies was performed. It objectified the presence of intraepithelial lymphocytes below 30%, corresponding to Marsh 0. To confirm the presence of CeD, given the high diagnostic suspicion, HLA testing was performed, which confirmed gluten intolerance.

The patient had advanced severe liver disease, with histological findings of abundant inflammatory infiltrate with mononuclear cells and frequent granulocyte, piecemeal necrosis, fibrosis in the porto-biliary spaces with a tendency to progress into septa, and neo-biliary canaliculi. The clinical phenotype of this patient (an obese male child), along with negative liver autoantibodies but a slight increase in gamma globulins, histological findings of inflammation, piecemeal necrosis, fibrosis, and without evidence of obstructive features on MRCP, did not allow a rapid diagnostic or a facile categorization. According to diagnostic criteria for AIH, the patient has a score highly suggestive of the diagnosis of AIH.

The main diagnostic considerations were autoantibody-negative autoimmune hepatitis associated to CeD and celiac hepatitis as a form of onset of CeD. Autoantibody-negative autoimmune hepatitis associated to CeD was the most appropriate diagnosis for this patient. The treatment introduced consisted of prednisone, azathioprine, and, not least, a strict GFD. The patient responded completely to this treatment. After one year, despite periods of inadequate adherence to the GFD and missed periodic examinations, the patient is on GFD and treatment with azathioprine for two more years and exhibits a very good general condition, normalized biological tests (transaminases, bilirubin, gamma-glutamyl transferase, gamma globulins, anti-tissue transglutaminase IgA antibodies, and anti-endomysial antibodies), and normal liver appearance at ultrasound examination but with a slight splenomegaly. The grade II/III splenomegaly observed at diagnosis was attributed to advanced massive chronic hepatitis with cirrhotic evolution. However, the persistence of mild splenomegaly after the normalization of liver morphology suggests that this finding might be related to the patient’s height, which is above the +3.71 SD for age. It is known that the spleen size can be slightly larger in taller individuals [36]. On the other hand, the evolution of splenomegaly in AIH under treatment with prednisone and azathioprine has not been thoroughly characterized in the current literature. In the absence of baseline splenic measurements before disease onset, ongoing patient monitoring is necessary.

## 4. Conclusions

The case presented illustrates AIH associated with CeD in a 12-year-old male obese patient, who presented with symptomatic elevated transaminases. Differentiating between autoantibody-negative AIH associated with CeD as an overlap syndrome and celiac hepatitis as an onset form of CeD was particularly challenging. CeD can be associated with a wide range of liver disorders, from mild increase in serum aminotransferases to more severe liver diseases, with elevated liver enzymes being the most common presentation. The pathogenesis is complex and involves multiple mechanisms, and while most liver abnormalities improve with a GFD, autoimmune liver disorders may require additional treatment. Regular monitoring of serum aminotransferases is recommended for celiac patients, and elevated serum aminotransferases in celiac patients warrant further evaluation for autoimmune predisposition, while screening for CeD in AIH patients could be clinically valuable.

The association of CeD with obesity adds further complexity to the case. The phenotype of CeD has evolved significantly over the past few decades, with clear trends towards older age at diagnosis, increased prevalence of non-classical and subclinical forms, and a shift from undernutrition to overweight and obesity. However, the relationship between malabsorption and overweight or obesity in CeD patients remains unclear. These changes are likely due to improved diagnostic methods, increased awareness, and possibly environmental factors. Understanding these trends is crucial for the more effective management and diagnosis of CeD across diverse populations.

## Data Availability

This article does not include any additional primary data besides the information already presented in the case report section.
